# Molecular dynamics of the host response to *Streptococcus pneumoniae* pneumonia in baboons

**DOI:** 10.1002/ame2.70079

**Published:** 2025-09-14

**Authors:** Bryan D. Kraft, Ashlee M. Valente, Ephraim L. Tsalik, Micah T. McClain, Marshall Nichols, Thomas W. Burke, Ricardo Henao, Erik J. Soderblom, J. Will Thompson, M. Arthur Moseley, Lori L. Hudson, Timothy Veldman, Olga M. Better, Mert Aydin, Anna Mazur, Karen E. Welty‐Wolf, Claude A. Piantadosi, Geoffrey S. Ginsburg, Christopher W. Woods

**Affiliations:** ^1^ Division of Pulmonary, Allergy, and Critical Care Medicine, Department of Medicine Duke University School of Medicine Durham North Carolina USA; ^2^ Durham Veterans Affairs Health Care System Durham North Carolina USA; ^3^ Division of Pulmonary and Critical Care Medicine, Department of Medicine Washington University School of Medicine Saint Louis Missouri USA; ^4^ Center for Infectious Disease Diagnostics and Innovation Duke University School of Medicine Durham North Carolina USA; ^5^ Division of Infectious Diseases, Department of Medicine Duke University School of Medicine Durham North Carolina USA; ^6^ Proteomics and Metabolomics Shared Resource Duke University School of Medicine Durham North Carolina USA; ^7^ All of Us Research Program, National Institutes of Health Bethesda Maryland USA

**Keywords:** animal models, bacterial pneumonia, gene expression profiling, Streptococcus pneumoniae, rapid diagnostic tests

## Abstract

**Background:**

Bacterial pneumonia remains a leading cause of morbidity and mortality worldwide despite the widespread availability of antibiotics. Novel pneumonia therapies and biomarkers are urgently needed to improve outcomes and advance personalized therapy. Using an established baboon model of *S. pneumoniae* pneumonia, we sought to characterize the temporal dynamics of pneumonia host responses to identify novel potential diagnostic and therapeutic molecular targets.

**Methods:**

We performed whole blood transcriptomics, unbiased proteomics, and peripheral cytokine measurements serially in baboons inoculated with *S. pneumoniae* (*n* = 23) or saline (*n* = 10) and modeled the peripheral blood host response using principal components analysis and complex sparse logistic regression. Differentially expressed genes were analyzed for pathway analysis.

**Results:**

Inoculated animals developed characteristic signs and symptoms of pneumonia. A 39‐gene signature was derived that classified *S. pneumoniae* infection with high accuracy (auROC 0.9 and 0.99 at 24 and 48 h post‐inoculation, respectively). Similar performance was observed for 48‐h biomarker signatures derived from peripheral blood plasma proteomic and cytokine measurements (both auROC >0.9). The gene signature retained strong diagnostic performance (auROC = 0.88) when transformed to human orthologs and applied to patients with acute respiratory illness (*n* = 34) or healthy controls (*n* = 20). Pathway analysis at 48 h identified down‐regulation of mitophagy and glucocorticoid signaling in peripheral blood.

**Conclusions:**

We report novel peripheral blood gene and protein expression signatures of *S. pneumoniae* pneumonia that could improve pneumonia diagnosis and found distinct pathways that may be amenable to modulation. Our findings illustrate how non‐human primate models of bacterial pneumonia can successfully translate biomarker discoveries to patients.

## Graphical abstract

Using an established, clinically relevant nonhuman primate model of *S. pneumoniae* pneumonia, this study aims to identify novel potential molecular diagnostic and therapeutic targets using peripheral blood bulk transcriptomic, unbiased proteomic, and cytokine measurements. The constructed models were highly accurate in classifying bacterial pneumonia in both nonhuman primates and adjudicated human cases. Pathway analysis identified glucocorticoid and mitophagy gene expression were down‐regulated in peripheral blood, suggesting these as novel targets for therapeutics.

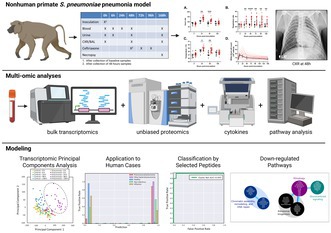

## 1 INTRODUCTION

Bacterial pneumonia is a leading cause of sepsis and significant morbidity and mortality worldwide, particularly for vulnerable populations such as children and aging adults.^1^, ^2^, ^3^ The prototypical bacterial pneumonia pathogen *Streptococcus pneumoniae* causes over 13 million pneumonia cases and over 700 000 pneumonia deaths per year.^2^ While bacterial pneumonia is a clinical diagnosis, platforms for detecting bacterial pathogens have significantly advanced over the last two decades, including urinary antigens,^4^ PCR,^5^ whole‐genome sequencing,^6^ and limited biomarkers of host responses such as cytokine production and acute phase reactants.^7^, ^8^, ^9^ However, there is still a significant gap in our understanding of pneumonia host responses and a need for better diagnostic and prognostic biomarkers for individualized pneumonia therapies.^10^

We and others^11^, ^12^, ^13^, ^14^, ^15^, ^16^, ^17^, ^18^ have utilized host gene expression profiles in peripheral blood to detect viral, bacterial, and fungal infections and characterize host responses. This approach assumes that high‐dimensional gene expression data offer higher precision to diagnose acute and chronic diseases.^19^ We have also demonstrated that analysis of orthogonal datasets such as transcriptomics, proteomics, and metabolomics provides a more comprehensive survey of the host response to infection.^20^, ^21^, ^22^

Non‐human primate (NHP) experimental models have played a fundamental role in investigations of sepsis and organ dysfunction over the past 30 years and have proven instrumental in bridging biological gaps between rodents and humans.^23^, ^24^, ^25^, ^26^, ^27^, ^28^ Baboons have an anatomy and lung physiology similar to humans, making them ideal models of human respiratory infection.^27^, ^29^, ^30^ We utilized an established and clinically relevant baboon model of *S. pneumoniae* pneumonia^24^ with defined inoculation and blood sampling to characterize host gene expression responses across multiple molecular analyte classes. Our findings suggest that this model is feasible for biomarker discovery relevant to human disease, can provide novel insights into the time course of the host response towards *S. pneumoniae* pneumonia, and may help identify novel potential therapeutic molecular targets.

## 2 METHODS

### 2.1 Animal experiments

**FIGURE 1:**
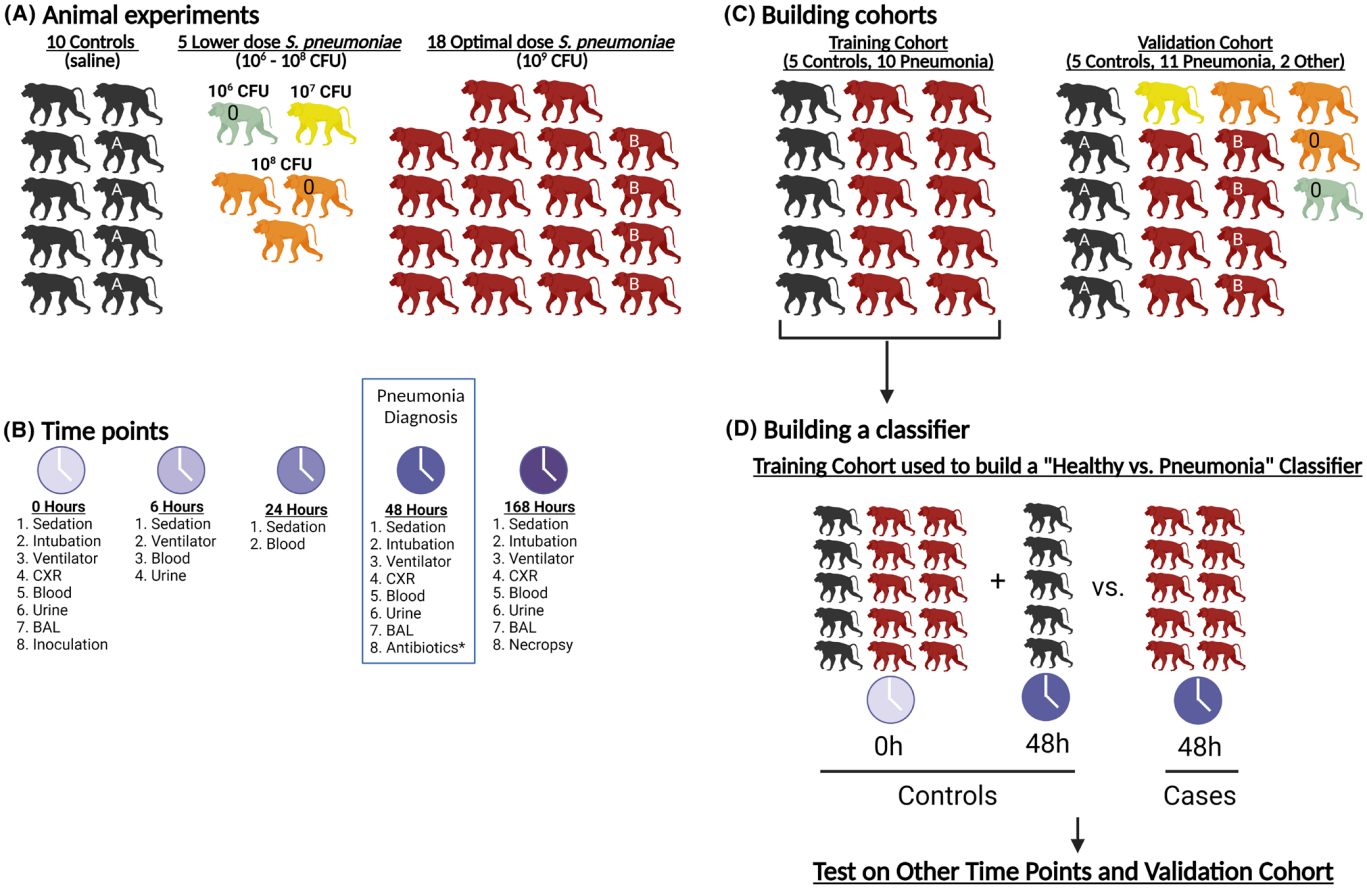
Experimental design and classifier construction. (A) Animal experiments. We performed *n* = 10 control experiments (saline; black animals), *n* = 5 lower dose *S. pneumoniae* experiments (10^6^, 10^7^, and 10^8^ CFU; green, yellow, and orange animals, respectively), and *n* = 18 optimal dose *S. pneumoniae* experiments (10^9^ CFU; red animals). A white “0” on a baboon indicates the baboon was inoculated at the dose shown but did not develop pneumonia. Four of the animals were used twice, first as controls, shown as “A”, and then as a 10^9^ CFU experimental animal, shown as “B”. (B) Time points. The sampling and clinical procedures performed are shown for each time point. The 48 h time point is boxed to signify when animals were considered to have pneumonia. *Antibiotics were administered daily for 3 days, starting at 48 h after collection of samples. (C) Building cohorts. The animals from (A) were divided into a training cohort (*n* = 5 controls and *n* = 10 pneumonia animals at 10^9^ CFU) and a validation cohort (*n* = 5 controls, *n* = 11 pneumonia animals at 10^7^–10^9^ CFU, and *n* = 2 “0” animals at 10^6^ or 10^8^ CFU that were inoculated but not meet pneumonia criteria at 48 h). (D) Building a classifier. The training cohort was used to build a classifier. The 48 h time point was the time at which a full assessment and pneumonia diagnosis was made and was the main time point used for constructing classification models. For each classification model, all animals at baseline (0 h) and all control animals at 48 h (48 h) were considered healthy controls, while all 10^9^ CFU animals at 48 h were considered *S. pneumoniae* pneumonia cases. This figure was created in BioRender.

Adult male colony‐bred baboons (*Papio cynocephalus*) from the Texas Biomedical Research Institute (San Antonio, TX) were housed in the Duke University Vivarium (Durham, NC) and handled in accordance with American Association for Accreditation of Laboratory Animal Care guidelines. The experimental protocol was approved by the Duke University Institutional Animal Care and Use Committee. The experimental procedures have been previously published.^24^ Briefly, the animals were sedated, intubated, and mechanically ventilated. *S. pneumoniae* (Serotype 19A‐7; ATCC, Manassas, VA) or sterile saline was delivered via bronchoscope into the lower left lobe and lingula. The inoculation doses were saline control (*n* = 10), 10^6^ CFU (*n* = 1), 10^7^ CFU (*n* = 1), 10^8^ CFU (*n* = 3), and 10^9^ CFU (*n* = 18) (Figure 1A). After 6 h, the animals were extubated and isolated. At 24 h, the animals were sedated and intubated and samples were collected. Ceftriaxone (1 g, Hospira Inc., Lake Forest, IL) was administered by intramuscular injection daily for 3 days starting after collection of the 48‐h samples. At 168 h, the animals were euthanized. Animals were evaluated daily for cough, nasal discharge, activity level, and oral intake. Chest radiographs were collected at 0, 48, and 168 h. Serial plasma and PAXgene Blood RNA samples were obtained at 0, 6, 24, 48, and 168 h (Figure 1B).

### 2.2 Definition of pneumonia and cohorts

Pneumonia has been defined previously,^24^ but, briefly, a diagnosis required a change in white blood cell count, isolation of *S. pneumoniae* from blood or bronchoalveolar lavage fluid, and signs or symptoms of pneumonia. For biomarker development, fifteen animals were designated as the training cohort (*n* = 5 control animals and *n* = 10 at 10^9^ CFU), and 18 animals were designated as the validation cohort (n = 5 control animals, *n* = 1 at 10^6^ CFU, *n* = 1 at 10^7^ CFU, *n* = 3 at 10^8^ CFU, and *n* = 8 at 10^9^ CFU) (Figure 1C). For the transcriptomic and proteomic statistical models, the uninfected group (“controls”) included baseline measurements in animals that later met pneumonia criteria as well as saline‐treated control animals at both baseline and 48 h. “Cases” consisted of 48‐h samples from animals given 10^9^ CFU meeting pneumonia criteria (Figure 1D).

### 2.3 Animal conservation

To conserve animals, two of the 10^9^ CFU‐inoculated animals within the validation cohort were from a similar study with only slight modifications to the protocol: *S. pneumoniae* was delivered bilaterally and divided between the lower lobes, lingula, and right middle lobe; and no samples were collected at 6‐ and 168‐h post‐inoculation. Additionally, four of the 10^9^ CFU animals were used first in a saline control experiment, and then once recovered (average time 65 days, range 35–112 days), were used again in a *S. pneumoniae* experiment.

### 2.4 Multi‐omic analyses

Methods used to conduct gene expression, proteomic, cytokine, and pathway analyses are described further in the Supporting Information.

## 3 RESULTS

### 3.1 Non‐human primate model of *S. pneumoniae* pneumonia

**FIGURE 2:**
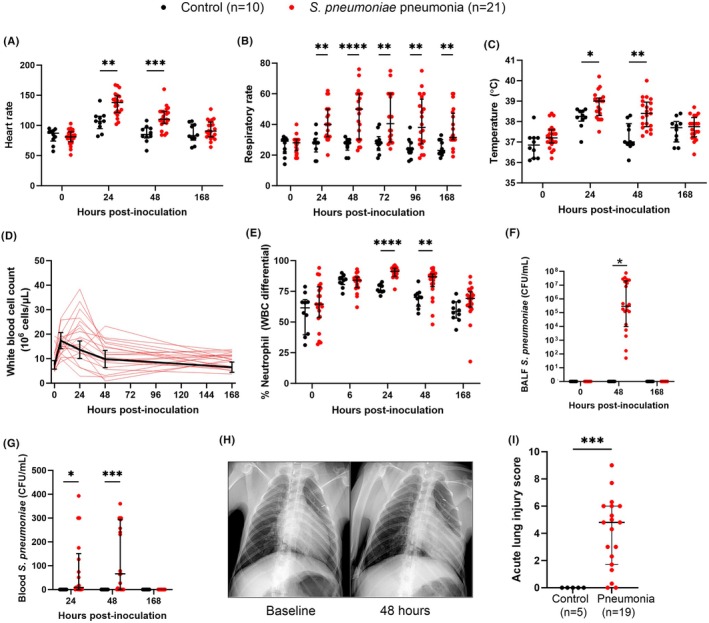
Nonhuman primate pneumonia model. Animals were inoculated with *S. pneumoniae* (red dots/lines) and met predefined pneumonia criteria (*n* = 21 total; doses included 10^7^ CFU [*n* = 1], 10^8^ CFU [*n* = 2], or 10^9^ CFU [*n* = 18]) or were given saline vehicle (black dots or line) as a control (*n* = 10). Animals with pneumonia developed tachycardia (A), tachypnea (B), and fever (C). (D) WBC counts are shown individually for the *S. pneumoniae*‐infected animals and grouped for control animals. Responses varied with some animals displaying leukocytosis and some leukopenia. (E) Peripheral blood percent neutrophils were significantly higher at 24 and 48 h post‐inoculation in *S. pneumoniae*‐infected animals. (F, G) The experimental strain of *S. pneumoniae* was isolated from BALF (F) and/or peripheral blood (G) at the time points shown. (H) Representative ventrodorsal chest radiograph from an animal at baseline and 48 h after inoculation with 10^9^ CFU, demonstrating left lower lobe infiltrate. (I) Blinded acute lung injury scores of lung tissue sections taken at necropsy (168 h) from *n* = 5 controls and *n* = 19 pneumonia animals. Individual scores for each animal are the sum of the scores (0–4) given for edema, cellular inflammation, fibrin, necrosis, and alveolar filling and averaged over three lung blocks. Statistical analysis for (A–C, E–G) is by GraphPad Prism 9 using 2‐way repeated measures ANOVA (or mixed‐effects model if there are missing values) with Sidak's post‐hoc test, and for (I) uses a Mann–Whitney test. *p < 0.05, **p < 0.01, ***p < 0.001, ****p < 0.0001. Horizontal lines show median and bars represent interquartile range. BALF, bronchoalveolar lavage fluid; CFU, colony‐forming units; WBC, white blood cell.

Thirty‐three NHPs were inoculated with *S. pneumoniae* (*n* = 1 at 10^6^ CFU, *n* = 1 at 10^7^ CFU, *n* = 3 at 10^8^ CFU, and *n* = 18 at 10^9^ CFU) or saline vehicle (*n* = 10) (Figure 1A,B). The clinical data from fifteen of these animal experiments was previously published in a dose‐finding pilot study where the optimal dose of *S. pneumoniae* was deemed to be 10^9^ CFU^24^ (Table S1). Following inoculation, the one animal given 10^7^ CFU, two of three animals given 10^8^ CFU, and all animals given 10^9^ CFU (*n* = 18) met criteria for pneumonia (total *n* = 21) and developed tachycardia, tachypnea, fever, change in white blood cell count, positive cultures, and/or infiltrates on chest radiograph by 48 h post‐inoculation, while control animals remained near baseline on most parameters (Figure 2). For subsequent analyses, animals meeting pneumonia criteria were divided into training (*n* = 15) and validation (*n* = 18) cohorts (Figure 1C).

### 3.2 Transcriptomic signatures accurately diagnose experimental *S. pneumoniae* infection in baboons

**FIGURE 3:**
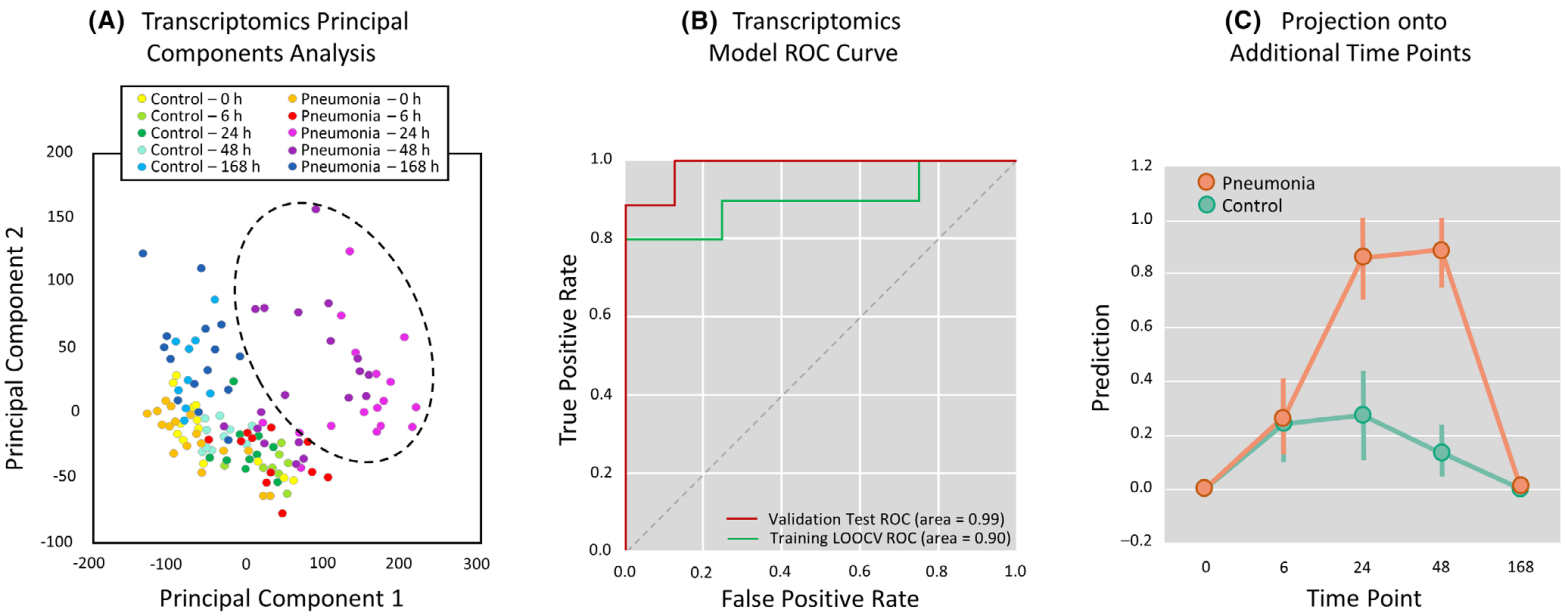
Blood host gene expression models accurately identify *S. pneumoniae* infection. (A) From the normalized RNAseq data, the first two principal components were computed and plotted. Each dot represents a single time point from a single animal, and dots are color‐coded according to time point and clinical pneumonia status. The black dotted circle represents clustering observed in the Pneumonia group for 24 and 48 h post‐inoculation. (B) Using the classification results of the training cohort (leave‐one‐out cross‐validation; LOOCV), we plot the false positive rate (FPR) versus true positive rate (TPR) of the training (green line) and validation (red line) cohorts at various classifier result thresholds, forming receiver‐operator characteristic (ROC) curves. The area under the curve (auROC) is computed as a metric of classification accuracy. (C) The classification model was also tested on all other time points to gain an understanding of how our signature would perform at various times in clinical presentation. The average and standard deviation of the classifier prediction score (*y*‐axis) is shown over time (*x*‐axis), with the orange line indicating prediction scores for animals meeting pneumonia criteria, and the green line indicating prediction scores for control animals. A classifier score of 1 would indicate a highly confident assignment to the pneumonia class, while a classifier score of 0 would indicate a highly confident assignment to the control class.

Whole blood collected at 0, 6‐, 24‐, 48‐, and 168‐h following inoculation was analyzed for RNA expression. Baboons' RNA data were of high quality and mapping rates (average of 65.13%) were sufficient for subsequent analysis. Normalized read counts were initially used for exploratory PCA that demonstrated clustering of the 24‐ and 48‐h time points among animals with pneumonia (Figure 3A). The top 100 most differentially expressed genes between pneumonia and control animals in the Training Cohort (Figure 1D) were then utilized to develop statistical classifiers using Elastic Net regularized logistic regression^31^ with LOOCV. The resulting mRNA classifier identified pneumonia with auROC = 0.90 using LOOCV classification (Figure 3B). The classification model was then tested on the Validation Cohort (Figure 1C) and classified pneumonia with an auROC = 0.99 (Figure 3B). The final signature consisting of 39 genes with non‐zero model weights was then applied to all available time points to mimic the variable times that patients present with acute illness (Figure 3C). There was no separation at 0 h (pre‐inoculation) and 6 h post‐inoculation when pneumonia criteria were not met, or at 168 h (post‐antibiotics) during the resolution phase of pneumonia. However, the model provided excellent classification accuracy at 24 h post‐inoculation (auROC = 0.9), suggesting an association between signature magnitude and acute illness.

### 3.3 Transcriptomic signatures accurately diagnose *S. pneumoniae* infection in humans

**FIGURE 4:**
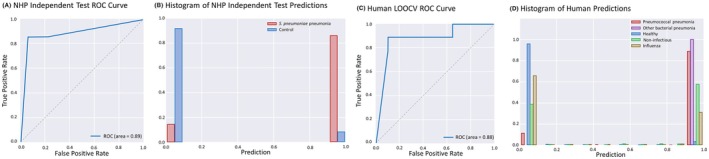
A sparse transcriptomic signature on an RT‐PCR platform accurately diagnoses bacterial infection. (A) Using the classification results of the independent test cohort of nonhuman primates, we plotted the false positive rate (FPR) versus true positive rate (TPR) to generate the receiver‐operator characteristic (ROC) curve. The area under the curve is computed as a metric of classification accuracy. (B) A normalized histogram of the classifier prediction scores in the nonhuman primates is shown for animals meeting pneumonia criteria (red bars) and for healthy animals (blue bars). A classifier score of 1 (*x*‐axis) would indicate a highly confident assignment to the pneumonia class, while a classifier score of 0 would indicate a highly confident assignment to the healthy class. (C) Using the LOOCV classification results of the human TLDA model, we plot the false positive rate versus true positive rate to generate the ROC curve. (D) A normalized histogram of the classifier prediction scores in humans is shown for pneumococcal pneumonia cases (red bars), other bacterial pneumonia (purple bars), healthy controls (blue bars), non‐infectious acute respiratory failure (green bars), and acute influenza infection (tan bars).

Having established a classifier signature for experimental NHP *S. pneumoniae* pneumonia, we then migrated the baboon signatures to an assay using human orthologs to test their potential use in human patients. The baboon signature was used to design a targeted TaqMan Low Density Array (TLDA) real‐time PCR experiment using assays for the human orthologs of the 39 NHP mRNAs. Fourteen signature transcripts and two reference transcripts had greater than 50% undetermined values in NHP samples (i.e. the measurement was unsuccessful or below quantitation), compared to 2 transcripts in human samples (Figure S1A). Further analysis showed that this attrition was directly related to the percent identity between the human TLDA assay and the original baboon gene transcript, with lower percent identity predicting non‐detection of RNA transcripts (Figure S1B). Despite these challenges in cross‐species translation, we still obtained high classification accuracy in NHP samples using human assays. The normalized expression of the remaining gene transcripts in the training cohort were utilized to develop a statistical classifier followed by validation in 18 baboons where it classified pneumonia with an auROC = 0.89 (Figure 4A,B).

We then sought to validate the RT‐PCR test on human subjects with *S. pneumoniae* pneumonia (*n* = 10), other bacterial pneumonia (*n* = 4), influenza (*n* = 11), non‐infectious acute respiratory illness (*n* = 9), and health controls (*n* = 20). The human cohort demographics are shown in Table S2. After deriving human‐specific weights for this signature, the test demonstrated an auROC = 0.88 in the human cohort comparing bacterial pneumonia with healthy controls (Figure 4C,D). The signature did not perform as well in the classification of influenza or non‐infectious respiratory failure, giving incorrect classifications of bacterial pneumonia.

### 3.4 Unbiased proteomics analysis yields highly accurate predictors of *S. pneumoniae* infection

**FIGURE 5:**
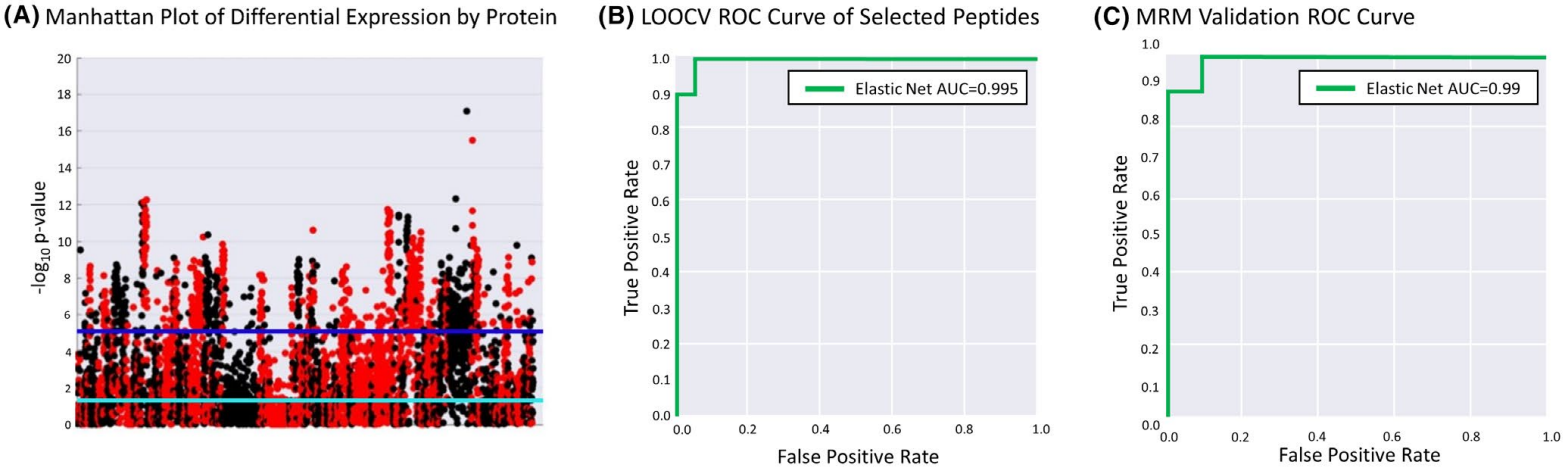
Unbiased and targeted proteomics offer diagnostic capability. (A) For each peptide quantified in the unbiased proteomics data, we computed a *p*‐value for the significance of differential expression between all uninfected samples (all baseline samples and control animals at 48 h) and all 48‐h pneumonia samples regardless of inoculum. We then illustrated the significant differential expression results using a modified version of a genome‐wide association study Manhattan plot. Each stack of points along the *x*‐axis in alternating red and black colors corresponds to a protein. Each point in the stack corresponds to a peptide measured for that protein. The position of a point on the *y*‐axis corresponds to the negative log of the *p*‐value for that peptide. Higher points indicate more significant differential expression. The light blue line corresponds to *p* = 0.05, and the dark blue line corresponds to the Bonferroni correction limit. (B) Using the selected peptides in the unbiased proteomics data, we constructed an Elastic Net classification model. Using the LOOCV results and varying thresholds of classifier predictions, we plot the false positive rate versus true positive rate to generate the receiver‐operator characteristic (ROC) curve. The area under the curve is computed and shown in the legend as a metric of classification accuracy. (C) MRM was tested in non‐immunodepleted plasma samples from the 15‐animal discovery cohort. An Elastic Net classification model was constructed. We plot the false positive rate versus true positive rate to generate the ROC curve. The areas under the curve (AUCs) are computed and shown as metrics of classification accuracy.

We then performed unbiased quantification of proteins in plasma via LC–MS/MS. The proteomics data were subject to differential expression analysis (Figure 5A) and supervised selection of the top 100 differentially expressed proteins. We then developed statistical classifiers using Elastic Net regularized logistic regression. LOOCV was used to determine hyperparameter settings and to estimate classification accuracy. Like the transcriptomic analyses, protein classifiers provided excellent LOOCV classification accuracy (auROC >0.95). To determine whether this approach would be feasible on a smaller, readily deployable platform, we down‐selected to 249 significant peptides comprising 76 proteins (Table S3) to design a targeted Multiple Reaction Monitoring (MRM) assay. To ensure the validity of the peptide selection approach, we re‐constructed our classification models in the unbiased dataset using only the down‐selected peptides and observed highly accurate classification of pneumonia and non‐pneumonia cases (auROC = 0.995, Figure 5B). We then performed targeted MRM assays to quantify plasma expression of these 249 peptides in all animals. However, classification accuracy was lower, with the Elastic Net model producing an independent test auROC = 0.82. To determine whether the decreased accuracy was technical in nature due to immunodepletion, non‐immunodepleted plasma samples from the discovery cohort underwent MRM to quantify expression followed by construction of classification models. The normalized peptide intensities for the 15 discovery animals were utilized to develop a statistical classifier using Elastic Net regularized logistic regression. LOOCV was used to determine optimal hyperparameter settings and an unbiased estimate of classification accuracy. The classification of pneumonia and non‐pneumonia cases improved with an auROC = 0.99 (Figure 5C).

### 3.5 Measurement of peripheral blood cytokines offers additional diagnostic potential

**FIGURE 6:**
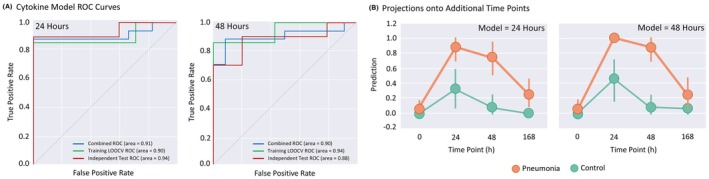
Cytokine levels differentiate bacterial pneumonia from health controls. (A) Using the classification results of the training (LOOCV) and independent test cohorts, we plotted the false positive rate (FPR) versus true positive rate (TPR) of the training, test, and combined cohorts at various classifier result thresholds, generating three receiver‐operator characteristic (ROC) curves. The area under the curve (“area”) is computed as a metric of classification accuracy. The model generated using cytokine data from 24 h is shown on the left, and the model generated using cytokine data from 48 h is shown on the right. (B) The classification models were also tested on all other time points to gain an understanding of how our signatures would perform at various points in clinical presentation. The average and standard deviation of the classifier prediction score (*y*‐axis) is shown over time (*x*‐axis) for animals meeting pneumonia criteria (orange line) and control animals (green line). A classifier score of 1 (*y*‐axis) would indicate a highly confident assignment to the pneumonia class, while a classifier score of 0 would indicate a highly confident assignment to the control class. The model generated using cytokine data from 24 h is shown on the left, and the model generated using cytokine data from 48 h is shown on the right.

Cytokine levels were measured for plasma samples at 0‐, 24‐, 48‐, and 168‐h post‐inoculation for all animals in the study using a NHP cytokine 24‐plex assay. Individual cytokine plots for control and 10^9^ CFU pneumonia animals are shown in Figure S2. Relative to controls, cytokines that were significantly elevated in the pneumonia cohort at 24 and/or 48 h after inoculation (evolution phase) were IL‐1β, IL‐6, IL‐15, IL‐16, IL‐17a, IP‐10, MCP‐1, MIP‐1β, and TARC; IL‐5 was significantly elevated at 168 h (resolution phase). Classifiers were constructed at both 24‐ and 48‐h time points using the designated training cohort with LASSO regularized logistic regression. Models based on cytokine data alone provided an excellent signal for *S. pneumoniae* pneumonia classification at both 24 and 48 h. Models at 24 and 48 h achieved auROC values of 0.9 and 0.94, respectively, while the independent test auROC values were 0.94 and 0.88, respectively (Figure 6A).

These models were also tested at all other time points and attained high levels of accuracy: the 24‐h model achieves an auROC of 0.92 at 48 h, and the 48‐h model achieves an auROC of 0.96 at 24 h (Figure 6B). Neither model identified a difference between pneumonia and healthy states at baseline or at 168 h during the resolution phase, 5 days after initiation of antibiotics.

### 3.6 Transcriptomic pathway analysis identifies key host response themes at 48 h post‐inoculation

**FIGURE 7:**
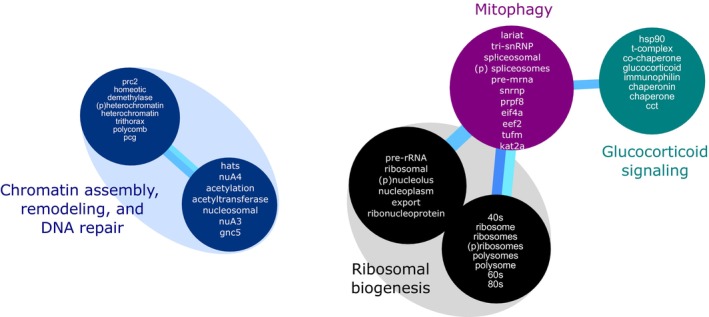
Pathway analysis of top down‐regulated genes. Pathway analysis was performed by COMPBio (see Section 2) for the top down‐regulated genes. The themes (individual circles) are grouped by color‐coding and/or color‐annotations. The size of the theme is proportional to its impact. Each theme contains a list of entities related to the input list. The entities and themes were assigned by COMPBio and the color‐coding and annotations were assigned by the authors.

Having identified the top differentially expressed gene transcripts in blood at 48 h post‐inoculation, we performed a transcriptomic pathway analysis using the COMPBio engine.^32^ The top 5 factors included 540 up‐regulated (Table S4) and 306 down‐regulated genes (Table S5). Pathway analysis identified significantly up‐regulated pathways (all *p* < 0.001) in peripheral blood, including glucose metabolism, lipolysis, antioxidant responses, cell proliferation, and innate immune responses related to pathogen‐receptor recognition and phagocytosis (Figure S3). Significantly down‐regulated pathways (all *p* ≤ 0.002) identified in peripheral blood included ribosomal biogenesis, mitophagy, glucocorticoid signaling, and chromatin remodeling and repair (Figure 7 and Figure S4).

## 4 DISCUSSION

We used a highly clinically relevant NHP model of *S. pneumoniae* pneumonia^24^ to investigate the time course of the host response in peripheral blood, and developed novel host biomarker signatures using transcriptomics, proteomics, and cytokine platforms. We further demonstrate strong performance of the NHP‐derived transcriptomic signature to classify human bacterial pneumonia cases and identify potentially clinically relevant pathways that may be useful pharmacologic targets. Overall, this work illustrates several findings that advance acute respiratory illness biomarker development: (1) the use of NHP models for biomarker discovery and translation to humans; (2) the importance of time series data; and (3) the use of rich orthogonal biological datasets to describe host responses to infection.

Non‐human primate models of infection have long served to improve our understanding of infection pathogenesis, host response, organ failure, and ultimately advance care for patients.^27^ Our *S. pneumoniae* pneumonia model in baboons closely resembles the human disease^24^ and has already helped translate novel therapies for acute respiratory failure to the clinical arena.^25^, ^28^ Here we report its use as a biomarker development tool to address a gap in pneumonia diagnostics.

We deployed a multi‐platform approach using transcriptomic, proteomic, and cytokine‐based models to develop highly predictive classifiers for *S. pneumoniae* pneumonia. Each platform performed well and classified animals with bacterial pneumonia during times of acute illness with high accuracy and precision. The models also correctly classified non‐infected animals, illustrating the use of such tools to rule out bacterial pneumonia. We focused our analyses on the 48‐h time point, which most closely approximates the time in which patients might seek medical attention, however, the 24‐h gene expression and cytokine models also classified animals correctly. Both models could serve as novel early detection tools to accelerate bacterial pneumonia diagnosis and treatment and ultimately improve pneumonia outcomes. The transcriptomic signatures seem especially well suited for near‐term clinical application given the recent development of mRNA multianalyte assay platforms,^33^, ^34^, ^35^, ^36^ though mixed mRNA/protein modalities may also be feasible.^37^

There is already compelling evidence that host response can diagnose infection based on pathogen class‐specific gene expression signatures.^11^, ^12^, ^13^, ^14^, ^15^, ^16^, ^17^, ^18^, ^38^ While the gene expression signatures we report herein can correctly distinguish pneumonia from non‐pneumonia, models generated exclusively using data from animals with *S. pneumoniae* pneumonia and healthy controls may not be useful to classify other acute respiratory illnesses. We therefore validated this gene expression signature using real‐time PCR in four adjudicated clinical scenarios: *S. pneumoniae* pneumonia, other bacterial pneumonia, acute influenza infection, and non‐infectious acute respiratory illness. Nearly all subjects with bacterial pneumonia, due to either *S. pneumoniae* or other bacteria, were classified as having acute bacterial pneumonia, suggesting host peripheral blood gene expression during acute *S. pneumoniae* pneumonia may closely resemble that of other bacterial pathogens. Furthermore, gene transcripts (or homologues) from our top 5 up‐regulated factors overlap with those of Tsalik, et al.^12^ who reported transcript classifiers in human subjects with acute bacterial pneumonia: CCR1, CPNE1, SERTAD3, CDKN2B (CDKN1A), GNG2 (GNG7), MTMR3 (MTMR1), NINJ2 (NINJ1), RPL6/12 (RPL28), TAF2 (TAF4), and YWHE/G (YWHAB). Classification for subjects with acute influenza infection or non‐infectious acute respiratory failure was less accurate, likely explained by the lack of inclusion of these groups in the training datasets or model building phase of classifier construction. Consequently, the derived classifier may be a representation of acute illness rather than bacterial pneumonia specifically. While more technically challenging, this underscores the rationale for including a variety of acute respiratory illnesses in the training datasets to produce a robust host classifier that may be more successfully deployed in a setting of diverse clinical presentations.^12^, ^39^

A competent host response to infection is likely influenced by the virulence of the bacterial strain studied. Following the introduction of the seven‐valent pneumococcal conjugate vaccine, the incidence of invasive pneumococcal infection due to *S. pneumoniae* serotype 19A, the serotype chosen for this study, increased significantly and now accounts for nearly half of all invasive pneumococcal infections in children.^40^, ^41^ Therefore, the gene signatures reported herein are reflective of invasive disease and may not be applicable to lung‐localized *S. pneumoniae* infections caused by less virulent serotypes. We hypothesize that the host response is serotype‐dependent, where invasive serotypes may suppress (or make ineffective) local production of cytokines and chemokines and recruitment of neutrophils and monocytes,^42^ but stimulate more cytokine and innate immunity gene expression in the peripheral blood.^43^ Signatures may also be affected by immune status^44^ and inoculum size.^24^, ^42^

Immunoaffinity depletion is considered a standard procedure in plasma proteomics research to enhance identification of lower abundance disease‐specific protein biomarkers.^45^ In our study, differences emerged in classifier accuracy between the immunodepleted plasma samples (worse classification) and the non‐depleted samples (better classification). This suggests the immunodepletion step may have removed high abundance proteins or proteins bound to the depleted high abundance proteins that were nevertheless important in classifying host response to bacterial pneumonia. This point should be considered by future proteomic studies of host response to infections.

Bacterial pneumonia causes acute lung injury with epithelial cell loss and mitochondrial damage.^46^, ^47^, ^48^ Clearance of damaged mitochondria by mitophagy is a critical lung injury resolution response^47^, ^49^ and may be impaired in non‐resolving acute respiratory distress syndrome.^48^ In this study, we used the peripheral blood transcriptome of baboons with *S. pneumoniae* pneumonia to perform pathway analysis and found that peripheral blood mitophagy was down‐regulated. The analysis specifically identified ABCE1 and TUFm which interact with PINK1,^50^, ^51^ and PRPF8, which regulates hypoxia‐induced mitophagy via alternative splicing.^52^ Because mitochondrial damage is evident in peripheral blood mononuclear cells of patients with sepsis,^53^ our findings suggest mitophagy may be dysregulated in *S. pneumoniae* pneumonia and may be a novel potential pharmacologic target. Pathway analysis also identified down‐regulation of ribosomal biogenesis, a major consumer of cellular energy, which may reflect cellular prioritization of energy usage during acute infection; although free ribonucleoprotein also coordinates cell stress responses suggesting this could represent a molecular sensor.^54^

The use of corticosteroids as a therapy for community‐acquired bacterial pneumonia is controversial, though some studies have found steroids accelerate pneumonia resolution^55^, ^56^ and improve survival,^57^ mechanistically linked to attenuating host inflammatory responses.^58^ Our pathway analysis found glucocorticoid signaling was down‐regulated in peripheral blood. This observation could provide proof‐of‐concept for why steroid therapy has been observed to benefit certain patients and deserves further consideration in future studies as bacterial pneumonia endotyping improves.^10^, ^59^

Our study has several limitations worthy of discussion. First, due to the lack of NHP viral respiratory illness data, we were unable to distinguish clearly between bacterial and viral infections in the human acute respiratory illness cases. While this was not the original intent of our study, future NHP studies could model influenza with *S. pneumoniae* bacterial super‐infection to address this. Second, we observed imperfect alignment between NHP and human orthologs. This may be, in part, because some of the human subjects may have had lung‐limited *S. pneumoniae* infection, rather than invasive disease, like our baboons. Third, our NHPs were heterogeneous and genetically diverse: While this may have weakened our ability to identify subtle biomarker signatures otherwise seen in a homogeneous animal cohort, it may also make our findings more generalizable to a diverse patient population. Furthermore, while large for a baboon study, and similar in size to prior human challenge experiments,^60^, ^61^, ^62^, ^63^, ^64^, ^65^ our sample size of thirty‐three animals may have been too small to sufficiently characterize pneumonia biomarker signatures in a heterogenous animal cohort; translation of our transcriptomic and proteomic findings into novel diagnostic tests would require validation in a larger sample size. Fourth, the single time point we used for pathway analysis does not inform the temporal host response and deserves further study. Fifth, our use of time point pairs at 0 h and 48 h in the control group for classifier training may not qualify as complete biological replicates, although this was done to conserve animals and control for experimental procedures. Sixth, we used a reference human genome for transcript alignment rather than a species‐specific reference genome. While this method may have allowed us to focus on human translation of the data, a lower mapping average (65.13%) may have meant that other key genes relevant to infection host response were not identified by the analysis. Finally, our inclusion of exposed but not infected animals that did not meet criteria for pneumonia as controls may have diluted our ability to observe differences between groups, though this was based on our prior human challenge studies.^61^, ^63^, ^64^

In conclusion, our preclinical NHP pneumonia model offers novel insights into the host response to *S. pneumoniae* infection and demonstrates the potential for NHP models to advance biomarker‐based assays in human populations with acute disease. The experimental and analytical models described herein provide a platform from which additional novel diagnostics may be developed and support future studies to better define and diagnose acute respiratory illnesses.

## AUTHOR CONTRIBUTIONS

**Bryan D. Kraft:** Data curation; formal analysis; investigation; methodology; visualization; writing – original draft; writing – review and editing. **Ashlee M. Valente:** Data curation; formal analysis; investigation; methodology; validation; visualization; writing – original draft; writing – review and editing. **Ephraim L. Tsalik:** Formal analysis; investigation; methodology; supervision; writing – original draft; writing – review and editing. **Micah T. McClain:** Formal analysis; investigation; methodology; supervision; writing – original draft; writing – review and editing. **Marshall Nichols:** Data curation; investigation; writing – review and editing. **Thomas W. Burke:** Formal analysis; investigation; methodology; supervision; validation; writing – review and editing. **Ricardo Henao:** Data curation; investigation; methodology; writing – review and editing. **Erik J. Soderblom:** Data curation; investigation; methodology; resources; writing – review and editing. **J. Will Thompson:** Data curation; investigation; methodology; resources; writing – review and editing. **M. Arthur Moseley:** Data curation; investigation; methodology; resources; writing – review and editing. **Lori L. Hudson:** Investigation; project administration; writing – review and editing. **Timothy Veldman:** Investigation; project administration; writing – review and editing. **Olga M. Better:** Data curation; investigation; writing – review and editing. **Mert Aydin:** Data curation; investigation; writing – review and editing. **Anna Mazur:** Data curation; investigation; writing – review and editing. **Karen E. Welty‐Wolf:** Conceptualization; data curation; funding acquisition; investigation; methodology; writing – review and editing. **Claude A. Piantadosi:** Conceptualization; data curation; investigation; methodology; supervision; writing – review and editing. **Geoffrey S. Ginsburg:** Conceptualization; funding acquisition; investigation; methodology; supervision; writing – review and editing. **Christopher W. Woods:** Conceptualization; data curation; investigation; methodology; supervision; validation; writing – review and editing.

## FUNDING INFORMATION

This study was funded by the Bill and Melinda Gates Foundation (Grant/Award Number: OPP1017554).

## CONFLICT OF INTEREST STATEMENT

The authors list the following disclosures (last 3 years): BDK reports consulting honoraria from Bluejay Diagnostics, Allucent, InflaRx, Verona Pharma, Eli Lilly, and GST Micro; and has patents granted or pending for “Compositions comprising mesenchymal stromal cells for the treatment of viral infections” and “Coronavirus antibodies and uses thereof”. ELT and GG previously held equity in Predigen and Biomeme and have patents granted or pending for Methods for Characterizing Infections and Methods for Developing Tests for the Same, Gene Expression Signatures Useful to Predict or Diagnose Sepsis and Methods of Using the Same, Methods to Diagnose and Treat Acute Respiratory Infections, and Biomarkers for the Molecular Classification of Bacterial Infection. ELT is currently employed by Danaher Inc. CWW and TWB consult for and holds equity in Biomeme and has patents pending or granted for “Methods to Diagnose and Treat Acute Respiratory Infections” and “Nasopharyngeal Protein Biomarkers of Acute Respiratory Virus Infection and Methods of Using Same”.

## ETHICS STATEMENT

All experimental procedures involving animals adhered to the ethical guidelines and welfare standards established by the Duke University Institutional Animal Care and Use Committee (protocol #A278‐08‐10 and #A271‐11‐10, PI: Piantadosi).

## Supporting information


Data S1.

## Data Availability

Data generated from this study will be shared by the lead contact upon request.
