# Supplementary material for: Molecular dynamics of the host response to Streptococcus pneumoniae pneumonia in baboons
Source: Animal Model Exp Med. 2025 Sep 14;8(10):1896–907. doi: 10.1002/ame2.70079 (PMC12660496; doi:10.1002/ame2.70079)
Supplement: Supplementary file 1 — Data S1. [file AME2-8-1896-s001.zip › ame270079-sup-0007-TableS1-S6@Supplemental File_R2.docx]

**SUPPLEMENTAL FILE**

**Molecular Dynamics of the Host Response**

**to *Streptococcus pneumoniae* Pneumonia in Baboons**

Bryan D. Kraft, MD^1-3^^,^, Ashlee M. Valente, PhD^4^, Ephraim L. Tsalik, MD, PhD^2,4,5^, Micah T. McClain, MD, PhD^2,4,5^, Marshall Nichols, MS^4^, Thomas W. Burke, PhD^4,5^, Ricardo Henao, PhD^4^, Erik J. Soderblom, PhD^6^, J. Will Thompson, PhD^6^, M. Arthur Moseley, PhD^6^, Lori L. Hudson, PhD^4^, Timothy Veldman, PhD^4^, Olga M. Better, BS^4^, Mert Aydin, MS^4^, Anna Mazur, BA^4^, Karen E. Welty-Wolf, MD^1,2^, Claude A. Piantadosi, MD^1,2^, Geoffrey S. Ginsburg, MD, PhD^7^, and Christopher W. Woods, MD, MPH^2,4,5^

^1^Division of Pulmonary, Allergy, and Critical Care Medicine, Department of Medicine, Duke University School of Medicine, Durham, North Carolina, 27710, USA

^2^Durham Veterans Affairs Health Care System, Durham, North Carolina, 27705, USA

^3^Division of Pulmonary and Critical Care Medicine, Department of Medicine, Washington University School of Medicine, Saint Louis, Missouri, 63110, USA, kraft@wustl.edu

^4^Center for Infectious Disease Diagnostics and Innovation, Duke University School of Medicine, Durham, North Carolina, 27710, USA

^5^Division of Infectious Diseases, Department of Medicine, Duke University School of Medicine, Durham, North Carolina, 27710, USA

^6^Proteomics and Metabolomics Shared Resource, Duke University School of Medicine, Durham, North Carolina, 27710, USA

^7^*All of Us* Research Program, National Institutes of Health, Bethesda, Maryland, 20892, USA

**SUPPLEMENTAL METHODS**

*Gene Expression Analysis*

We quantified mRNA gene expression in peripheral blood via bulk RNA-Sequencing (RNAseq) from whole blood collected in PAXgene Blood RNA tubes (PreAnalytiX) and stored at -80C. Total RNA was isolated using the PAXgene Blood miRNA kit, and RNA quality and yield were assessed using Agilent Bioanalyzer and Nanodrop spectrophotometer. RIN values of ≥7 were used for sequencing library preparation. Rarely, lower RIN samples were used depending on availability of additional PAXgene Blood RNA sample for re-extraction, and resulting sequencing data were flagged for additional data quality review. Abundant globin transcripts were depleted using GLOBINClear Human Globin RNA Reduction Kit (Ambion) and RNAseq libraries prepared using the Illumina TruSeq RNA Library Prep Kit. Illumina 50x50 paired end sequencing at 25 million reads per sample was performed (Expression Analysis (EA), Durham, NC). Because there was minimal transcriptional annotation information and no reference transcriptome available at the time of this analysis for the model species (*Papio cynocephalus*), and we are primarily interested in genes with a direct human ortholog, we followed published recommendations for comparing reference-based RNAseq mapping methods for non-human primate data^1^ and used the human transcriptome as a reference for mapping and quantification. Sequence data was mapped directly to the hg19 build of the human transcriptome with the Bowtie2 algorithm. Read quality analysis was performed on the raw data using FastQC version 0.10.1^2^. Quality trimming and adapter clipping were performed using Trimmomatic version 0.25^3^, trimming trailing bases below quality 20, clipping Illumina adapters, and discarding clipped reads shorter than 25 bp. FastQC was used to re-assess the integrity of the clipped reads prior to subsequent mapping and analysis. Reads whose mates were discarded due to quality trimming and length constraints were removed from the fastq files used for mapping. The Illumina iGenomes UCSC hg19 human reference genome and annotation was used as a reference, downloaded March 2013. To generate a fasta file of transcripts for a reference transcriptome, the BEDTools “getfasta” utility version 2.17.0^4^ was used to extract the transcript sequences specified by coordinates in the Ensembl GTF file, from the reference genome sequence. Both the Ensemble reference annotation and genomic sequence files were those included in the iGenomes download. Clipped reads were mapped to the hg19 genome and transcriptome using Bowtie2 version 2.0.6.^5^. Default parameter settings were used for all methods. SAM/BAM conversions, sorting, indexing, and marking of PCR duplicates were performed with SAMtools version 0.1.18^6^ and Picard software package version 1.83 ([http://picard.sourceforge.net](http://picard.sourceforge.net/)). Mappings were quantified with eXpress, normalized with the upper-quartile read counts within each sample, and log-transformed (**Figure S5**). The normalized read counts for the 15 animals designated as the training cohort were subject to exploratory principal components analysis (PCA) and differential expression testing. The top 100 differentially expressed genes were utilized to develop statistical classifiers using Elastic Net regularized logistic regression^7^. Leave-one-out-cross-validation (LOOCV) was used to determine hyperparameter settings.

*Gene Expression Signature Validation*

From the sparsity-inducing classification model of the RNAseq data, we obtained a final signature by using all transcripts with non-zero coefficients. This signature was used to design a targeted TaqMan Low Density Array (TLDA; Thermo Fisher Scientific) real-time PCR experiment, run on all NHP baseline and 48-hour post-inoculation samples, and the following human samples: *S. pneumoniae* pneumonia (n=10), other bacterial pneumonia (n=4), influenza (n=11), non-infectious acute respiratory illness (n=9), and healthy controls (n=20). The TLDA probes were designed using the human reference for optimal translation to human samples, and specific assays are shown in **Table S6**. The targeted assay data were subject to quality assurance and normalization with reference genes^8^. The normalized expression for the 15 animals designated as the training cohort were utilized to derive new model weights for the Elastic Net regularized logistic regression, using LOOCV to determine optimal hyperparameter settings. The classification model was then tested on the NHP validation cohort to determine classification performance. Human-specific model weights and classification accuracies were obtained using nested LOOCV to set model hyperparameters and obtain unbiased accuracy estimates.

*Unbiased Proteomics*

Unbiased quantification of plasma proteins via LC-MS/MS was performed as previously described^9^ for the 0- and 48-hour time points for the 15 animals in the training cohort. The resulting proteomics data were subject to unbiased variance filtering and differential expression analysis. The top 100 differentially expressed proteins were used to develop statistical classifiers with Elastic Net^7^ regularized logistic regression. LOOCV was used to determine hyperparameter settings and generate an unbiased estimate of classification accuracy.

*Targeted Proteomics Validation*

We then selected 249 peptides comprising 76 proteins to design a targeted Multiple Reaction Monitoring (MRM) assay based on several parameters: results of the classification modeling; differential expression testing results; lack of technical variability; ability to measure multiple peptides; homology with human proteins; and favorable chemical characteristics for targeted quantification (**Figure S6**). The targeted MRM assay was performed as previously described^9^ and utilized to quantify plasma expression of selected peptides in all animals. The normalized peptide intensities for the 15 animals designated as the training cohort were utilized to develop a statistical classifier using Elastic Net regularized logistic regression. LOOCV was used to determine optimal hyperparameter settings. The classification models were then tested on the validation cohort to determine classification performance.

*Cytokine Measurements*

Cytokines were measured in plasma samples at 0-, 24-, 48-, and 168-hours post-inoculation using the NHP Cytokine 24-plex Kit on the MESO QuickPlex SQ 120 instrument (Meso Scale Discovery). Data were processed by discarding of out-of-range values, log transformation, standardization, and adjustment for baseline to correct for high inter-animal variability. Single-time point, single-analyte comparisons, and classifiers were constructed at 24- and 48-hour time points using the designated training cohort with LASSO regularized logistic regression. For each statistical model, LOOCV was employed to select optimal hyperparameters. Independent classification accuracy was assessed on the test cohort.

*Adjustment for Multiple Comparisons*

The Benjamini-Hochberg procedure was used in the multi-omic analyses to adjust p-values to account for multiple hypothesis testing, as described^10^.

*Pathway Analysis*

Pathway analysis was performed using the upregulated and downregulated genes of the top 5 factors identified in the peripheral blood transcriptomic datasets at 48 hours post-inoculation. Genes and fold-change were inputed into a knowledge engine called “Comprehensive Multi-omics Platform for Biological Interpretation” (COMPBio), as described^11^. P-value ≤0.05 and normalized enrichment score (NEScore) ≥1.3 were accepted as significant.

**TABLE S1: Animal groups**

| **Animal #** | **Dose (CFU)** | **Published^a^** | **Met pneumonia criteria** | **Cohort** | **Notes** |
| --- | --- | --- | --- | --- | --- |
| 16876 | Saline | Yes | No | Training |  |
| 19759 | Saline | Yes | No | Training |  |
| 26044 | Saline | Yes | No | Training |  |
| 27369 | Saline | Yes | No | Training |  |
| 28575 | Saline | No | No | Training |  |
| 29671-A | Saline | No | No | Validation | No necropsy |
| 29705-A | Saline | No | No | Validation | No necropsy |
| 29946 | Saline | No | No | Validation |  |
| 30221-A | Saline | No | No | Validation | No necropsy |
| 30236-A | Saline | No | No | Validation | No necropsy |
| 16911 | 10^6^ | Yes | No | Validation |  |
| 17366 | 10^7^ | Yes | Yes | Validation |  |
| 17449 | 10^8^ | Yes | Yes | Validation |  |
| 19471 | 10^8^ | Yes | No | Validation |  |
| 26010 | 10^8^ | Yes | Yes | Validation |  |
| 18041 | 10^9^ | Yes | Yes | Training |  |
| 25417 | 10^9^ | Yes | Yes | Training |  |
| 26041 | 10^9^ | Yes | Yes | Training |  |
| 26236 | 10^9^ | Yes | Yes | Training |  |
| 26418 | 10^9^ | Yes | Yes | Training |  |
| 27182 | 10^9^ | Yes | Yes | Training |  |
| 28293 | 10^9^ | No | Yes | Validation | Died at 48 hours^b^ |
| 28301 | 10^9^ | No | Yes | Training |  |
| 29083 | 10^9^ | No | Yes | Validation |  |
| 29223 | 10^9^ | No | Yes | Training | Modified protocol^c^ |
| 29308 | 10^9^ | No | Yes | Validation |  |
| 29613 | 10^9^ | No | Yes | Training |  |
| 29671-B | 10^9^ | No | Yes | Validation | Conserved |
| 29697 | 10^9^ | No | Yes | Training | Modified protocol^c^ |
| 29705-B | 10^9^ | No | Yes | Validation | Conserved |
| 30146 | 10^9^ | No | Yes | Validation |  |
| 30221-B | 10^9^ | No | Yes | Validation | Conserved |
| 30236-B | 10^9^ | No | Yes | Validation | Conserved |

^a^Pneumonia data from these animal experiments was previously published^12^.

^b^This animal was euthanized early at 48 hours due to meeting humane endpoints (refractory hypoxemia and shock), at which time necropsy was performed.

^c^Necropsy data are not reported for these animals due to modifications made to the protocol.

**TABLE S2: Human acute respiratory illness cases and controls**

| **Demographics** | ***S. pneumoniae* pneumonia**  **(n=10)** | **Other bacterial pneumonia^a^**  **(n=4)** | **Acute influenza infection**  **(n=11)** | **Non-infectious acute respiratory failure^b^**  **(n=9)** | **Healthy Controls**  **(n=20)** |
| --- | --- | --- | --- | --- | --- |
| **Age, mean (range)** | 50 (19-76) | 63 (59-69) | 28 (18-69) | 66 (47-87) | 19 (18-20) |
| **Sex (% male)** | 30% | 75% | 46% | 56% | 45% |
| **Race (%)** |  |  |  |  |  |
| White | 30.0% | 50.0% | 36.4% | 33% | 65.0% |
| Black | 70.0% | 50.0% | 18.2% | 67% | 0.0% |
| Asian | 0.0% | 0.0% | 27.3% | 0% | 15.0% |
| American Indian/ Alaska Native | 0.0% | 0.0% | 9.1% | 0% | 5.0% |
| Multiple races | 0.0% | 0.0% | 9.1% | 0% | 15.0% |
| **Ethnicity (%)** |  |  |  |  |  |
| Non-Hispanic | 100% | 100% | 55% | 100% | 90% |
| Hispanic | 0% | 0% | 0% | 0% | 0% |
| Unknown | 0% | 0% | 45% | 0% | 10% |

^a^Includes the following bacterial pathogens: *Pseudomonas aeruginosa* (n=1), *Streptococcus viridans* group (n=1), *Staphylococcus aureus* (n=1), *Pasteurella multocida* (n=1).

^b^Includes the following etiologies: congestive heart failure (n=8), renal failure (n=1).

**TABLE S3: List of top 76 plasma proteins differentially expressed at 48 hours**

| **Proteins [*Papio anubis*]** |
| --- |
| actin- alpha skeletal muscle |
| alpha-1-acid glycoprotein 1 |
| alpha-2-macroglobulin isoform X2 |
| alpha-N-acetylglucosaminidase |
| apolipoprotein D isoform X2 |
| apolipoprotein(a) |
| attractin-like |
| beta-hexosaminidase subunit beta |
| C4b-binding protein beta chain isoform X2 |
| cartilage acidic protein 1 isoform X2 |
| cholesteryl ester transfer protein isoform X1 |
| cholinesterase isoform X1 |
| coagulation factor V |
| coagulation factor X precursor |
| coagulation factor XI isoform X2 |
| coagulation factor XIII B chain-like |
| collectin-11 isoform X1 |
| complement C1r subcomponent-like protein isoform X1 |
| complement factor H-related protein 5 |
| coronin-1A |
| creatine kinase M-type |
| di-N-acetylchitobiase |
| exostosin-like 2 |
| fibulin-1 |
| galectin-3-binding protein |
| glutathione peroxidase 3 |
| hepatocyte growth factor-like protein isoform X1 |
| insulin-like growth factor I isoform X2 |
| insulin-like growth factor-binding protein 3 |
| insulin-like growth factor-binding protein 6 |
| intercellular adhesion molecule 1 |
| interleukin-1 receptor type 2 |
| kallistatin |
| keratin- type I cytoskeletal 14 |
| keratin- type I cytoskeletal 9 |
| keratin- type II cytoskeletal 2 epidermal |
| leukotriene A-4 hydrolase isoform X1 |
| L-lactate dehydrogenase A chain |
| lumican |
| lysosome-associated membrane glycoprotein 2 isoform X2 |
| lysozyme C precursor |
| mannan-binding lectin serine protease 1 isoform X1 |
| mannan-binding lectin serine protease 2 |
| mannosyl-oligosaccharide 1-2-alpha-mannosidase IA isoform X1 |
| myomesin-2 |
| neural cell adhesion molecule 1 isoform X14 |
| neural cell adhesion molecule L1-like protein isoform X3 |
| pancreatic secretory trypsin inhibitor |
| peptidase inhibitor 16 isoform X1 |
| peptidyl-prolyl cis-trans isomerase B |
| periostin isoform X2 |
| periostin isoform X6 |
| phosphatidylinositol-glycan-specific phospholipase D isoform X2 |
| pigment epithelium-derived factor |
| plasma kallikrein |
| plasma serine protease inhibitor |
| plastin-2 |
| pyruvate kinase PKM isoform X3 |
| scavenger receptor cysteine-rich type 1 protein M130 |
| selenoprotein P |
| serotransferrin |
| sulfhydryl oxidase 1 isoform X1 |
| thyroxine-binding globulin isoform X2 |
| transforming growth factor-beta-induced protein ig-h3 isoform X2 |
| trehalase isoform X1 |
| triosephosphate isomerase |
| ubiquitin-40S ribosomal protein S27a |
| vasorin |
| vinculin isoform X4 |
| vitamin K-dependent protein C isoform X1 |
| vitamin K-dependent protein S |
| vitamin K-dependent protein Z precursor |
| von Willebrand factor |
| xaa-Pro dipeptidase isoform X2 |
| xaa-Pro dipeptidase-like |
| zinc-alpha-2-glycoprotein |

**TABLE S6: TaqMan Low Density Array (TLDA) probes used for human translation**

| **TaqMan Assay ID** | **Gene Target Name** |
| --- | --- |
| Hs01106866_m1 | MS4A4A |
| Hs00154728_m1 | DECR1^a^ |
| Hs00356576_m1 | NINJ2 |
| Hs00415588_m1 | GRXCR1 |
| Hs00175188_m1 | CTSC |
| Hs01561006_m1 | FKBP5 |
| Hs00194524_m1 | RTN2 |
| Hs00198935_m1 | MARCO |
| Hs00984403_m1 | SLC28A1 |
| Hs00972326_m1 | TRAP1^a^ |
| Hs99999905_m1 | GAPDH^b^ |
| Hs01092524_m1 | LDLR |
| Hs00373758_m1 | MS4A6E |
| Hs00914120_m1 | F5 |
| Hs01027047_m1 | OTUB2 |
| Hs00360669_m1 | CD177 |
| Hs00831148_s1 | LGALS8-AS1 |
| Hs01634996_s1 | FCGR2B |
| Hs00168719_m1 | PPIB^a^ |
| Hs00269693_s1 | C3AR1 |
| Hs00269605_m1 | FCGR2C; FCGR2B |
| Hs00173145_m1 | DNASE1L1 |
| Hs00892591_m1 | EMR1 |
| Hs00396596_m1 | TBX20 |
| Hs02330328_s1 | SOCS3 |
| Hs00609162_m1 | EXT1 |
| Hs00172806_m1 | TOP3A |
| Hs01861627_g1 | TPM4 |
| Hs00218083_m1 | FAM63A |
| Hs01868673_s1 | CCDC70 |
| Hs00211643_m1 | RAB10 |
| Hs00191956_m1 | FPGS |
| Hs00944403_m1 | CNNM3 |
| Hs00420495_m1 | MYCL |
| Hs01123468_m1 | DIDO1 |
| Hs01042825_s1 | TSPYL1 |
| Hs00171699_m1 | WNT7A |
| Hs00233509_m1 | CD1C |
| Hs00367777_m1 | RGS3 |
| Hs01020652_m1 | DCAF8 |
| Hs01056538_m1 | KLHL21 |
| Hs00180312_m1 | MLLT3 |
| Hs00360269_m1 | VAMP2 |
| Hs00197392_m1 | TM9SF1 |

^a^Normalization control

^b^Manufacturing technical control

**SUPPLEMENTAL REFERENCES**

1. Benjamin, A.M., Nichols, M., Burke, T.W., Ginsburg, G.S., and Lucas, J.E. (2014). Comparing reference-based RNA-Seq mapping methods for non-human primate data. BMC Genomics *15*, 570. 10.1186/1471-2164-15-570.

2. Andrews, S. (2010). FASTQC. A quality control tool for high throughput sequence data. <http://www.bioinformatics.babraham.ac.uk/projects/fastqc>.

3. Lohse, M., Bolger, A.M., Nagel, A., Fernie, A.R., Lunn, J.E., Stitt, M., and Usadel, B. (2012). RobiNA: a user-friendly, integrated software solution for RNA-Seq-based transcriptomics. Nucleic Acids Res *40*, W622-627. 10.1093/nar/gks540.

4. Quinlan, A.R., and Hall, I.M. (2010). BEDTools: a flexible suite of utilities for comparing genomic features. Bioinformatics *26*, 841-842. 10.1093/bioinformatics/btq033.

5. Langmead, B., and Salzberg, S.L. (2012). Fast gapped-read alignment with Bowtie 2. Nat Methods *9*, 357-359. 10.1038/nmeth.1923.

6. Li, H., Handsaker, B., Wysoker, A., Fennell, T., Ruan, J., Homer, N., Marth, G., Abecasis, G., Durbin, R., and Genome Project Data Processing, S. (2009). The Sequence Alignment/Map format and SAMtools. Bioinformatics *25*, 2078-2079. 10.1093/bioinformatics/btp352.

7. Zou H, H.T. (2005). Regularization and variable selection via the elastic net. Journal of the Royal Statistical Society: Series B (Statistical Methodology) *67* (*2*), 301-320.

8. Livak, K.J., and Schmittgen, T.D. (2001). Analysis of relative gene expression data using real-time quantitative PCR and the 2(-Delta Delta C(T)) Method. Methods *25*, 402-408. 10.1006/meth.2001.1262.

9. Burke, T.W., Henao, R., Soderblom, E., Tsalik, E.L., Thompson, J.W., McClain, M.T., Nichols, M., Nicholson, B.P., Veldman, T., Lucas, J.E., et al. (2017). Nasopharyngeal Protein Biomarkers of Acute Respiratory Virus Infection. EBioMedicine *17*, 172-181. 10.1016/j.ebiom.2017.02.015.

10. Benjamini Y, H.Y. (1995). Controlling the false discovery rate: a practical and powerful approach to multiple testing. Journal of the Royal Statistical Society: Series B (Methodological) *57*, 289-300.

11. Ma, C., Storer, C.E., Chandran, U., LaFramboise, W.A., Petrosko, P., Frank, M., Hartman, D.J., Pantanowitz, L., Haritunians, T., Head, R.D., and Liu, T.C. (2021). Crohn's disease-associated ATG16L1 T300A genotype is associated with improved survival in gastric cancer. EBioMedicine *67*, 103347. 10.1016/j.ebiom.2021.103347.

12. Kraft, B.D., Piantadosi, C.A., Benjamin, A.M., Lucas, J.E., Zaas, A.K., Betancourt-Quiroz, M., Woods, C.W., Chang, A.L., Roggli, V.L., Marshall, C.D., et al. (2014). Development of a novel preclinical model of pneumococcal pneumonia in nonhuman primates. Am J Respir Cell Mol Biol *50*, 995-1004. 10.1165/rcmb.2013-0340OC.
