# Supplementary material for: Molecular dynamics of the host response to Streptococcus pneumoniae pneumonia in baboons
Source: Animal Model Exp Med. 2025 Sep 14;8(10):1896–907. doi: 10.1002/ame2.70079 (PMC12660496; doi:10.1002/ame2.70079)
Supplement: Supplementary file 1 — Data S1. [file AME2-8-1896-s001.zip › Figure caption.docx]

**Figure S1: Signature Attrition Migrating from RNA-Seq to TLDA.** (*A*) We noted significant proportions of undetermined values (i.e. measurement was unsuccessful) and below quantitation values (i.e. Cq value > 35) in several probes within non-human primate samples. The proportion of samples with values below the limits of TLDA quantitation are shown in the bar charts for both non-human primate and human samples. The genes on the y-axis are sorted according to proportion beyond usable limits. The 39 signature transcripts are shown in black font and white shading and the 5 reference transcripts are shown in white font and black shading. (*B*) In order to determine whether the attrition was related to percent identity between the human TLDA probe and the baboon gene, we computed the percent identity for all probes. The bivariate distribution of the percent identity and percent undetermined values is shown in a contour plot. Darker color indicated a larger number of observations in the region of the contour plot. We also computed the Pearson correlation coefficient between these two values, and found a significant negative correlation: Lower percent identity was significantly correlated with higher percent undetermined.

**Figure S2: Peripheral cytokines.** Plasma cytokine levels are shown for individual control animals (black circles) or pneumonia animals (10^9^ CFU) (grey circles) at 0, 24, 48, and 168 hours after inoculation. Statistical analysis is by GraphPad Prism 9 using 2-way repeated measures ANOVA (or mixed-effects model if there are missing values) with Sidak’s post-hoc test. *P<0.05, **P<0.01, ***P<0.001, ****P<0.0001. Horizontal lines show median and bars represent interquartile range.

**Figure S3: Pathway analysis of top up-regulated genes.** Pathway analysis was performed by COMPBio (see *Methods*) for the top up-regulated genes. The themes (individual circles) are grouped by color-coding and/or color-annotations. The size of the theme is proportional to its impact. Each theme contains a list of entities related to the input list. The entities and themes were assigned by COMPBio and the color-coding and annotations were assigned by the authors.

**Figure S4: Bar graph of enriched down-regulated biological pathways.** Pathway analysis was performed by COMPBio (see *Methods*) for the top down-regulated genes. The themes were assigned by COMPBio and the color-coding and annotations were assigned by the authors.

**Figure S5: RNA-Seq Data Processing.** Sequence data was subject to quality assessment and trimming, and then mapped directly to the hg19 build of the human transcriptome with the Bowtie2 algorithm. The resulting mappings were quantified with eXpress, normalized with the upper-quartile read counts within each sample, and log-transformed. The left graphic illustrates the processing procedure, and the histograms show the numbers of samples within ranges of percentage of reads surviving quality control before mapping (top), and mapping rates to the hg19 reference transcriptome (bottom).

**Figure S6: Peptide Selection Procedure for MRM Assay.** For all identified proteins in the experiment, we identify a human ortholog for each protein by maximal percent identity BLAST, simulate a tryptic digest, compute *in silico* molecular properties, and pull the differential expression results and model weights from the statistical analysis. From this full list, we then discard all peptides longer than 20 amino acids, all peptides containing methionine, all peptides below 80% sequence identity (human vs. baboon), and all peptides above the 95^th^ percentile for experimental technical variability. Using this list of peptides, we then discard the protein if there is only a single peptide remaining. Of the remaining proteins we discard any proteins if fewer than 50% of their represented peptides were significantly differentially expressed or had a non-zero coefficient in classification. With this final list of proteins and representative peptides, we select 3-4 peptides for each protein in the list sorted by: p-value, classification model weight, percent sequence identity, fold change, and technical variability. This figure was created in BioRender.
