# Supplementary figures and images for: Molecular dynamics of the host response to Streptococcus pneumoniae pneumonia in baboons
Source: Animal Model Exp Med. 2025 Sep 14;8(10):1896–907. doi: 10.1002/ame2.70079 (PMC12660496; doi:10.1002/ame2.70079)

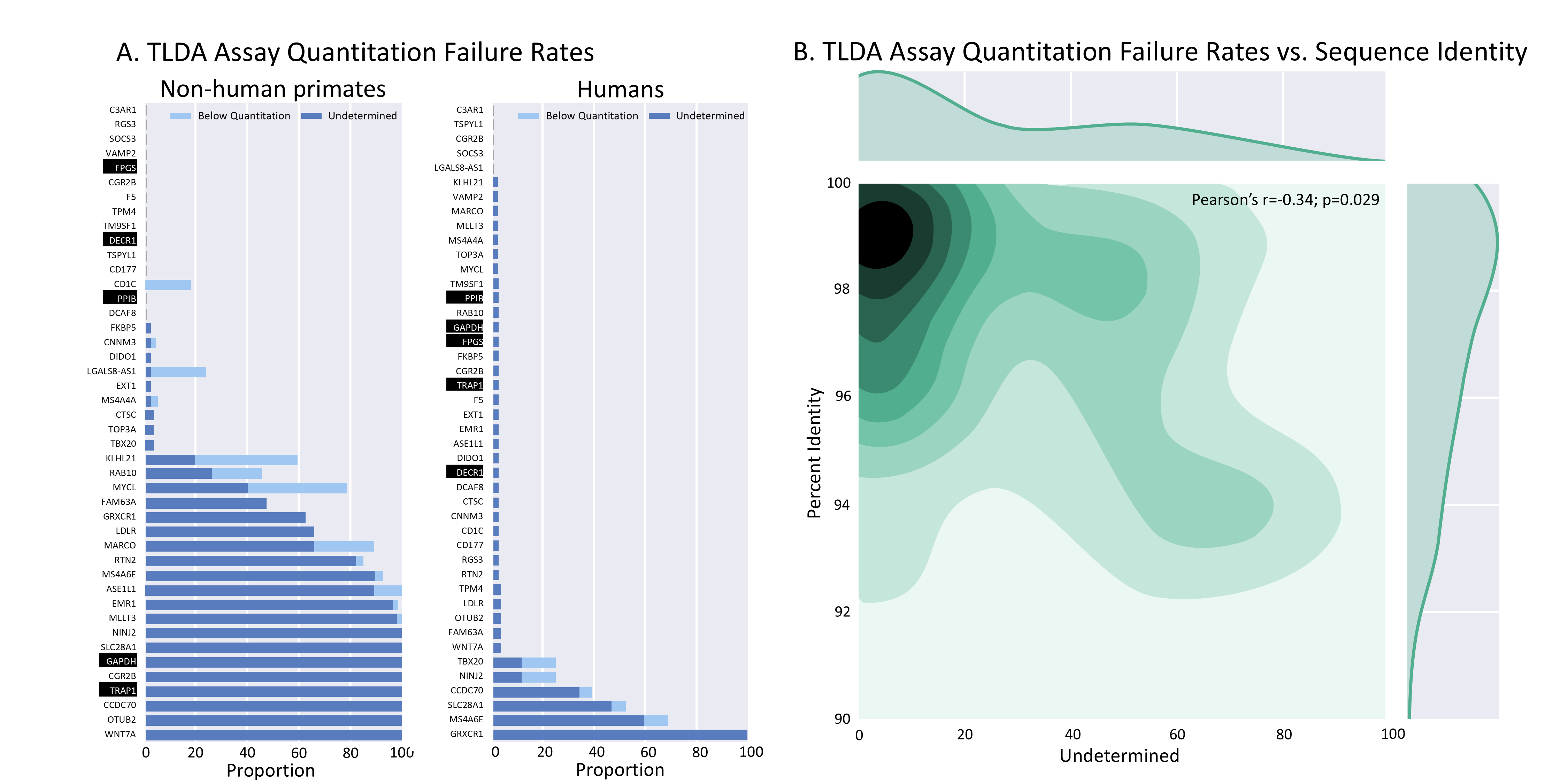

Supplement: Supplementary file 1 — Data S1. [file AME2-8-1896-s001.zip › ame270079-sup-0001-FigureS1@Figure S1_TLDA failure.tif]

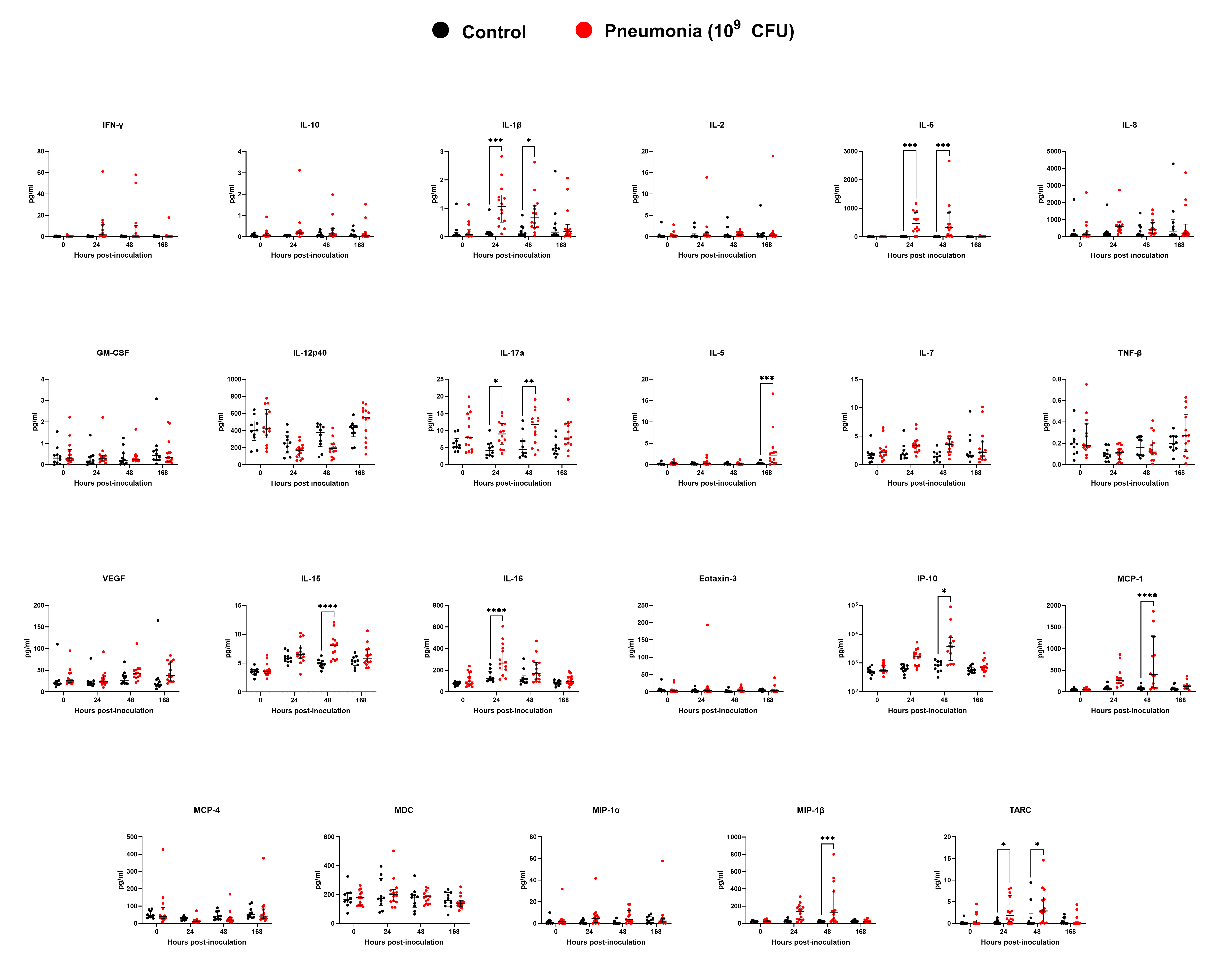

Supplement: Supplementary file 1 — Data S1. [file AME2-8-1896-s001.zip › ame270079-sup-0002-FigureS2@Figure S2_Cytokines.tif]

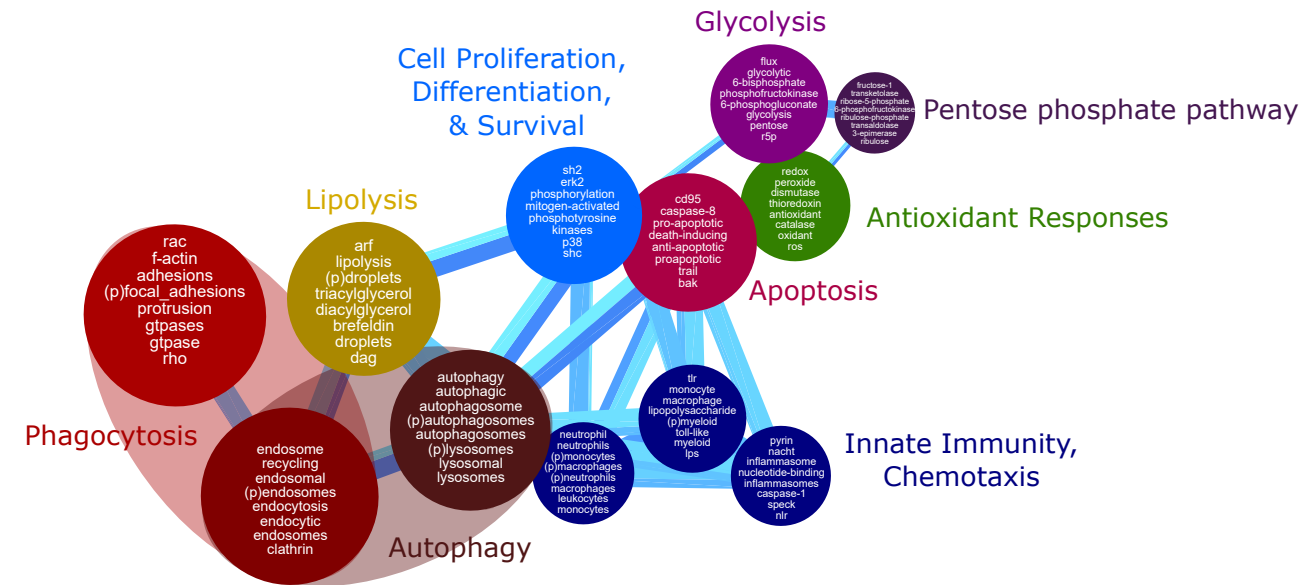

Supplement: Supplementary file 1 — Data S1. [file AME2-8-1896-s001.zip › ame270079-sup-0003-FigureS3@Figure S3_Upregulated COMPBio.pdf]

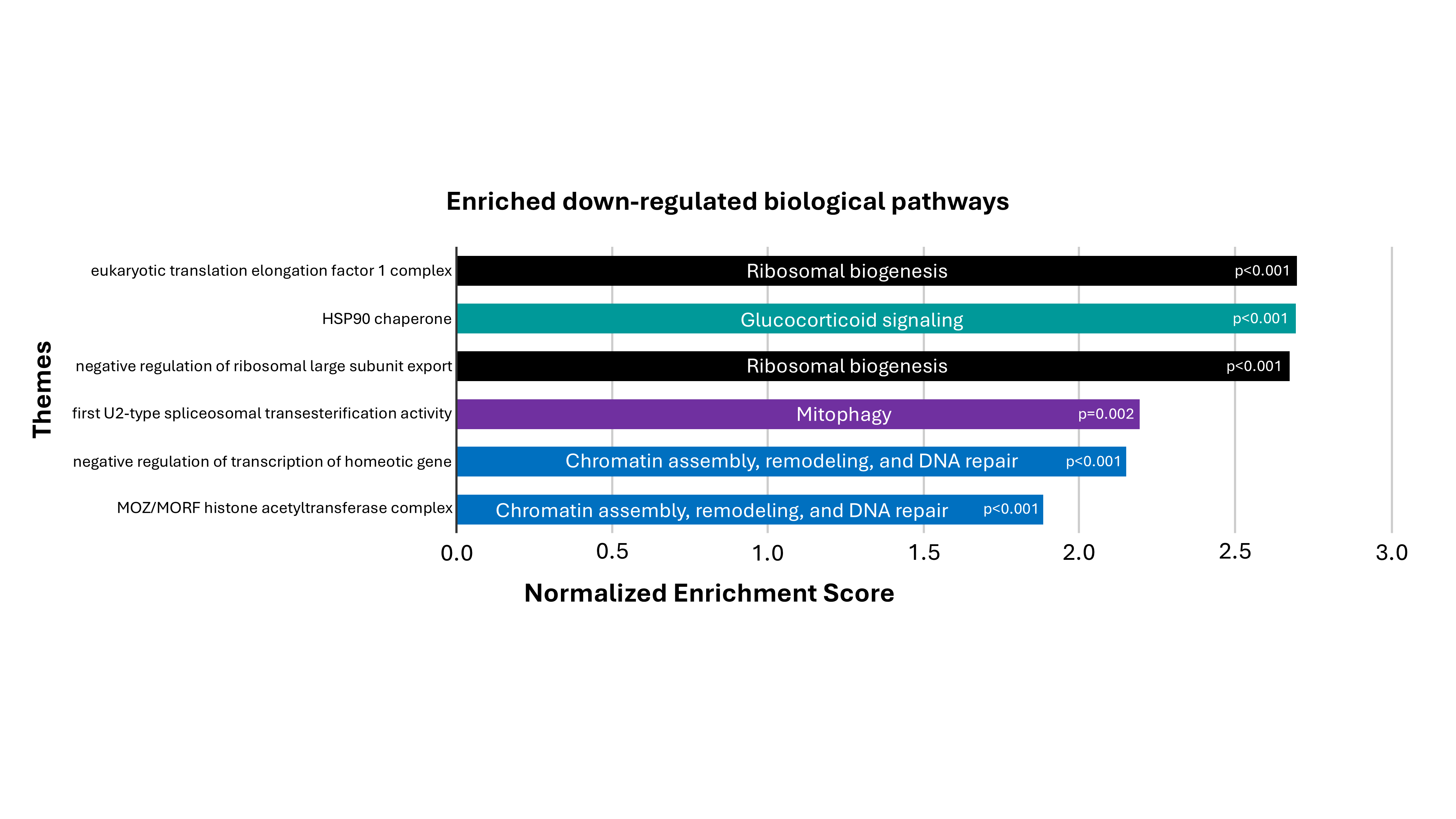

Supplement: Supplementary file 1 — Data S1. [file AME2-8-1896-s001.zip › ame270079-sup-0004-FigureS4@Figure S4_Bargraph of enriched biological pathways.tif]

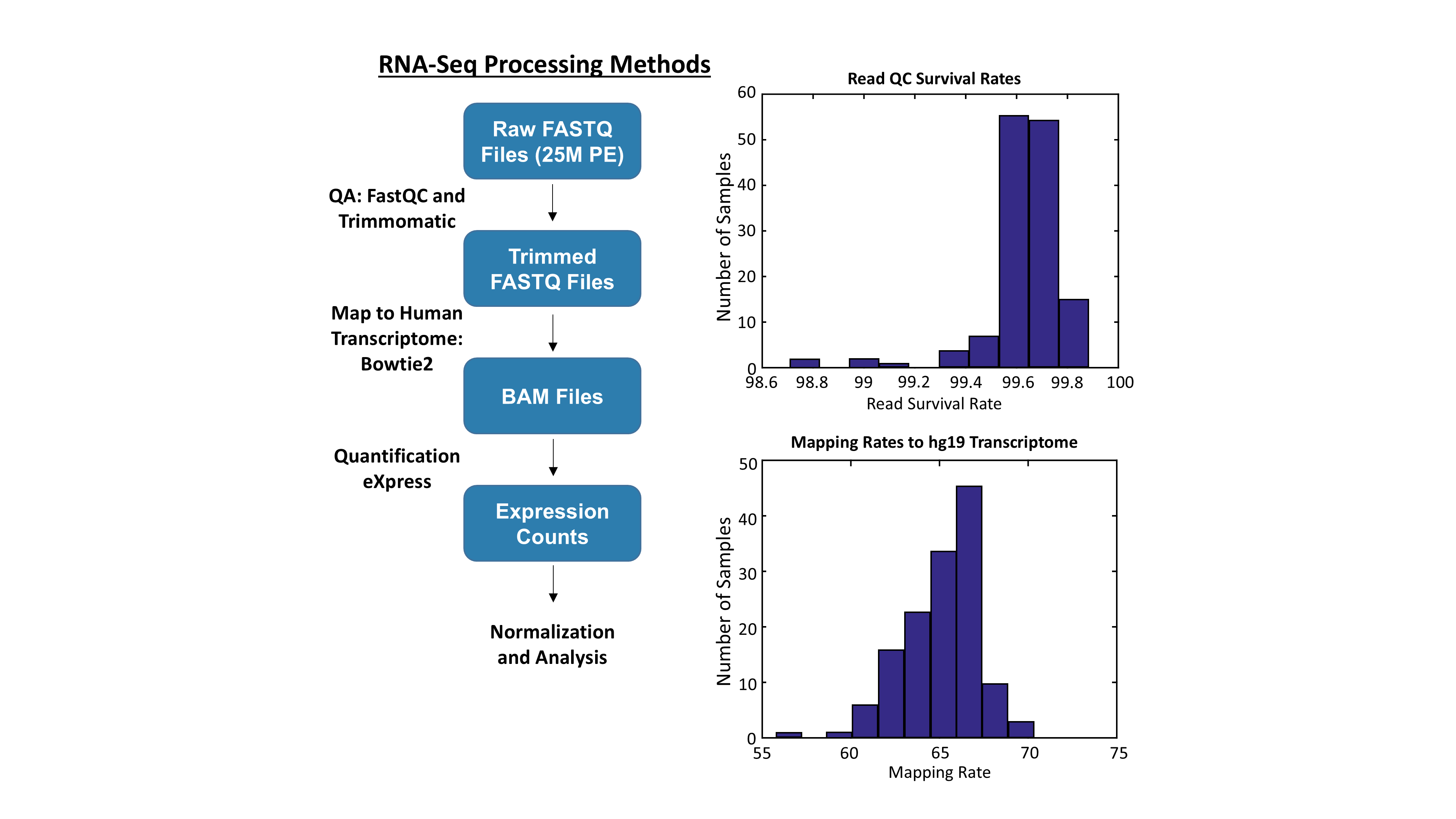

Supplement: Supplementary file 1 — Data S1. [file AME2-8-1896-s001.zip › ame270079-sup-0005-FigureS5@Figure S5_RNA processing.tif]

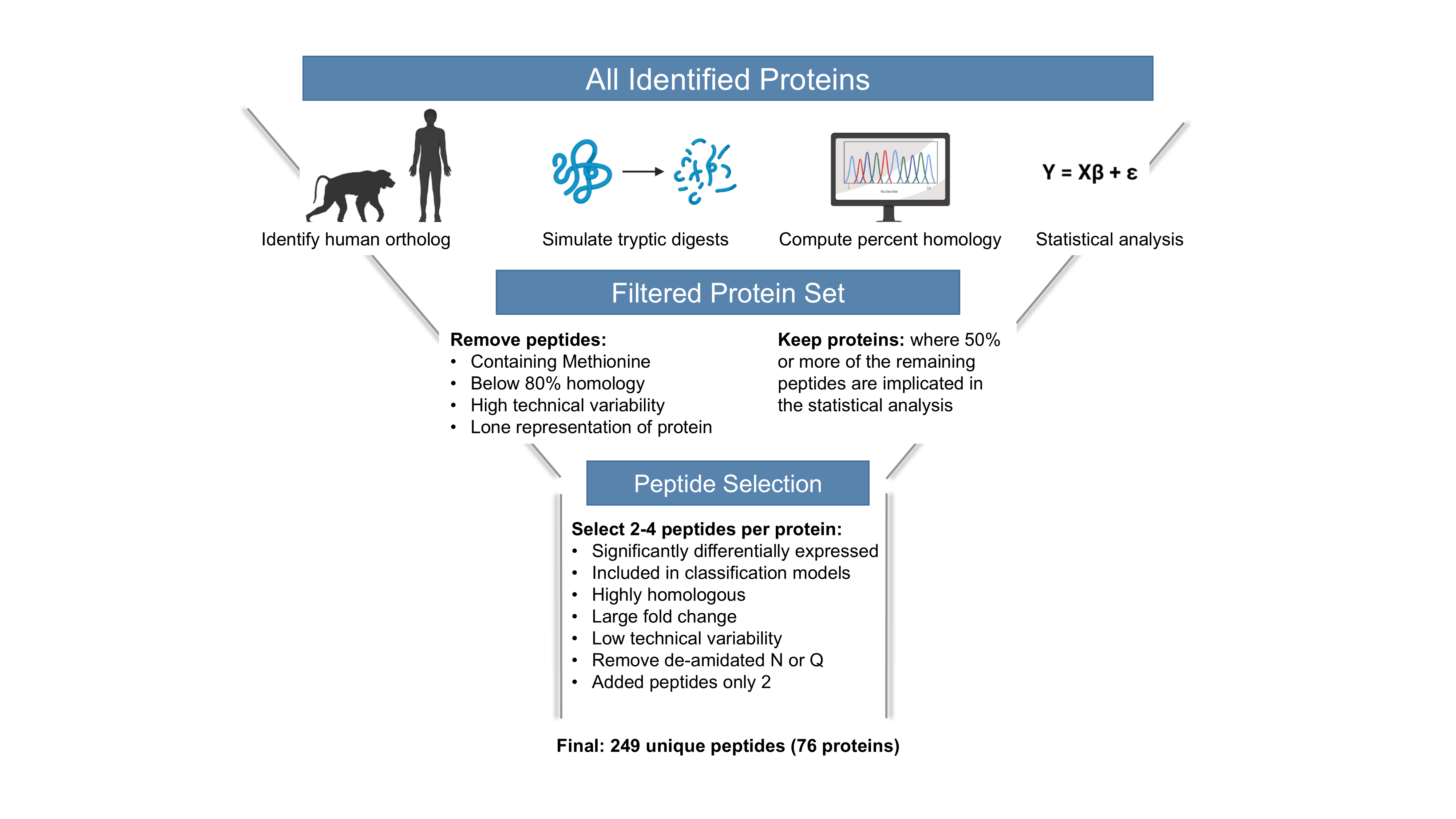

Supplement: Supplementary file 1 — Data S1. [file AME2-8-1896-s001.zip › ame270079-sup-0006-FigureS6@Figure S6_MRM analysis.tif]
